# Surface Modifications Induced by High Fluxes of Low Energy Helium Ions

**DOI:** 10.1038/srep09779

**Published:** 2015-04-28

**Authors:** İrem Tanyeli, Laurent Marot, Daniel Mathys, Mauritius C. M. van de Sanden, Gregory De Temmerman

**Affiliations:** 1 FOM-Institute DIFFER, Dutch Institute For Fundamental Energy Research, Edisonbaan 14, 3439MN, Nieuwegein, The Netherlands; 2 Department of Physics, University of Basel, Klingelbergstrasse 82, CH-4056 Basel, Switzerland; 3 Centre of Microscopy, University of Basel, Klingelbergstrasse 50/70, CH-4056 Basel, Switzerland; 4 ITER Organization, Route de Vinon sur Verdon, CS 90 046- 13067 St Paul Lez Durance Cedex, France

## Abstract

Several metal surfaces, such as titanium, aluminum and copper, were exposed to high fluxes (in the range of 10^23^ m^−2^s^−1^) of low energy (<100 eV) Helium (He) ions. The surfaces were analyzed by scanning electron microscopy and to get a better understanding on morphology changes both top view and cross sectional images were taken. Different surface modifications, such as voids and nano pillars, are observed on these metals. The differences and similarities in the development of surface morphologies are discussed in terms of the material properties and compared with the results of similar experimental studies. The results show that He ions induced void growth and physical sputtering play a significant role in surface modification using high fluxes of low energy He ions.

Surface structuring by energetic ion bombardment has been widely studied and considered as an efficient surface processing technique, since it is fast, cost effective and various types of material can be processed by this technique. This so-called ion beam sputtering technique is attributed to the removal of atoms from the surface due to the impact of energetic ions.^1^ Both experimental and theoretical studies have been conducted for a wide range of conditions, such as ion species, ion energy, surface temperature and angle of ion bombardment.^1^,^2^,^3^ As a particular case, the interaction of helium ions with metal surfaces, especially with tungsten, has long been studied extensively because of helium production in fusion reactors.^4^,^5^,^6^,^7^ More recently, significant surface modifications were observed on tungsten under low-energy He ion irradiation, with ion energies below the threshold for damage creation, and investigated as a function of surface temperature, ion flux and exposure time.^8^,^9^,^10^,^11^ These studies revealed the formation of a fine nanostructure exhibiting a high porosity of up to 90% and high light absorption.^12^,^13^,^14^ The size of those nanostructures and thickness of the nanostructured layer could be controlled by surface temperature and plasma exposure time, respectively.^15^ After these results on tungsten, investigations were extended to other metals. Similar nanostructure formation has been observed for molybdenum, nickel and iron surfaces under low energy He ion irradiation.^15^,^16^,^17^ Although a clear explanation of why some metals can be modified more easily than others is still missing, one could categorize these metals with respect to their crystal structure; except nickel, all of these metals have body centered cubic (bcc) type crystal structure.

Due to their controllable growth and porous structure, helium induced nanostructured surfaces appear to have a great potential to be used for various applications requiring high surface area and high light absorption, such as photo electrochemical water splitting for example.^18^,^19^,^20^ Indeed, an enhancement in the photocatalytic activity has been reported for nanostructured WO_3_, prepared by low-energy helium ion exposure and followed by annealing.^21^

In this study, we explore the effect of low-energy helium ion exposure of several metal surfaces, such as titanium (Ti), aluminum (Al) and copper (Cu). The choice of those metals was driven by the aforementioned application of photocatalytic properties of these metals in oxidized form. The influence of the different crystal structures on helium-plasma irradiation induced surface modification is studied and results are compared with both theoretical and experimental studies in literature.

## Effect of low energy He ions on surface modification of metals

Helium is a chemically inert gas and has almost zero solubility in metals, but can diffuse rapidly through the metal surface because of its relatively small size. These features of helium lead to bubble formation underneath the surface at crystallographic defects. Experimental and theoretical studies have shown that helium irradiation induced damages could be obtained even in the absence of displacement damage and native defects.^9^,^13^,^22^

Theoretical works conducted on tungsten and iron agree on a qualitative description of the formation and growth of clusters (or bubbles).^23^,^24^ Interstitial helium atoms are very mobile and tend to coalesce to form interstitial clusters. Both the interstitial He atoms and clusters can act as traps for incoming He atoms, which indicates a self-trapping ability of He. Once an interstitial He cluster reaches a sufficient size, it punches out a metal self-interstitial and forms a relatively immobile helium-vacancy cluster. Helium diffusion is required for nucleation of bubbles and their growth.^24^ In the case of negligible ion radiation damages, helium diffusion is dominated by interstitial migration and for higher temperatures (>0.5 *T_m_*, where *T_m_* is the melting temperature) the interstitially diffusing He atoms are mainly trapped by thermal vacancies, since the concentration of thermal vacancies tends to increase with temperature.^24^ There are 12 tetrahedral and 6 octahedral, 8 tetrahedral and 4 octahedral, 4 tetrahedral and 2 octahedral interstitial sites existing in metals with bcc, face centered cubic (fcc) and hexagonal close packed (hcp) type crystal structure, respectively.^25^ Since the interstitial sites could assist the He diffusion in the metal lattice, one could expect that helium trapping would be easiest in bcc type metals and less likely in closely packed metals. In addition to these self-interstitials in the metal, He ions could induce extra interstitials as mentioned above, which assist He diffusion and consequently nucleation, in the metal even if their energies are well below the knock-on energy for displacement damage. Besides, detrapping has to be taken into account during the discussion of helium trapping in the metals. The substitutional detrapping energies for bcc type metals listed in table 1 indicate that release of He atoms in a metal lattice is hardly expected from these metals. The formation and migration energies of He interstitials for different metals are compared in table 1.^26^,^27^,^28^,^29^,^30^,^31^,^32^ Although the values do not seem to differ much from each other, slightly higher formation and migration energies of hcp metal from the ones for fcc metals could indicate that He diffusion and clustering processes could be slower for hcp metals. Hence, one might expect that hcp-type metals will be less prone to He ion-induced morphology changes.

The experimental results have showed that nanobubble formation near the surface is necessary but not sufficient to give rise to nanostructure formation.^33^ The nanostructuring on tungsten surface is achieved for a surface temperature range of ~0.25 <*T*/*T_m_*<0.55.^8^ The upper boundary of the temperature range for nanostructure formation is defined by rapid surface diffusion, which could lead to surface smoothing and eventually disappearance of the nanostructure.^8^,^34^ The slow bubble growth rate is considered as the limiting factor at low temperature. Similar nanostructuring has been observed in the experimental studies conducted on Mo and Fe for intermediate temperatures (0.3 - 0.5 *T_m_*).^15^,^17^ The relation between swelling rate of helium bubbles and *T*/*T_m_* has already been reported.^34^ Relying on both experimental and theoretical studies, the temperature ranges worth to study for surface modification of different metals could be determined in advance.

## Methods

Polycrystalline titanium (99.99% purity, Goodfellow), copper (≥ 99.95% purity, Salomon’s Metalen) and aluminum (95.90% purity, Salomon’s Metalen) samples were exposed to pure helium plasma in Pilot-PSI, a high-flux linear plasma generator.^35^ The plasma is generated by a thermal plasma (cascaded arc) source and confined by an axial magnetic field. More detailed information about the experimental setup can be found elsewhere.^35^ The magnetic field was set to 0.2 T during our experiments. The plasma density profile has a Gaussian shape and the maximum ion flux to the surface was in the range of 2-7x10^23^m^−2^s^−1^. The samples were clamped on a water cooled target holder by a ring made from molybdenum. In order to have a better thermal contact, a Grafoil® layer was inserted between the sample and the target holder. The samples are negatively biased to control the ion energy, which is calculated with respect to plasma potential and sheath entrance voltage. Further details about ion energy calculation can be found in Ref 36.

Polycrystalline samples, which are 20 mm in diameter and 1 mm in thickness, were mechanically polished with SiC grinding papers and followed by 3 and 1 µm diamond and 0.05 µm alumina suspensions. The metals that we worked with have different levels of hardness, so that different polishing recipes have been followed. For Ti and Cu samples we used 320 to 2400 grit SiC papers and for Al samples 500 to 2400 grit SiC papers. The mirror finish polished samples were cleaned with a basic procedure, acetone, ethanol, de-ionized water in ultrasonic bath and for easy rinsing a further bath with ethanol repeated at the end.

During plasma exposure, the peak temperature was measured by a multiwavelength pyrometer (FMPI SpectroPyrometer, FAR Associates), which measures in the wavelength range of 900–1600 nm. In addition, an infrared camera (FLIR A645 SC) was used to measure the 2D surface temperature profile and was also used in case of temperatures lower than the detection limit of the pyrometer.

The surfaces were analyzed by high resolution scanning electron microscopy (SEM, Hitachi S-4800 field emission at 5 kV) and atomic force microscopy (Bruker Dimension Edge AFM ) in order to investigate the modifications after plasma exposures. For cross sectional imaging, the samples were prepared by focused ion beam (Dual Beam FIB/SEM) milling method.

## Results

### Titanium

**Figure 1:**
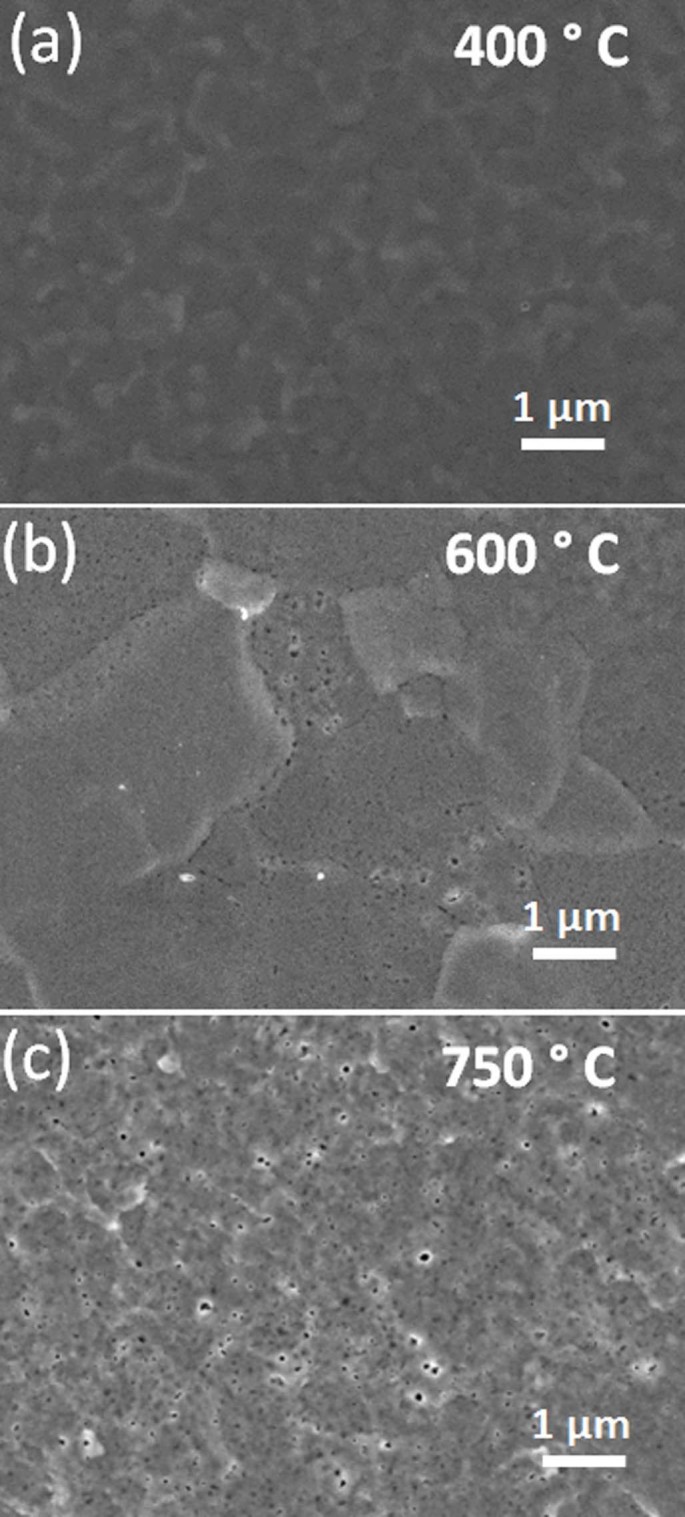
SEM images of Ti samples which are exposed to helium plasma with the surface temperature of 400°C (0.35 T_m_), 600°C (0.45 T_m_) and 750°C (0.53 T_m_) for 10 minutes: (i)-(iii) (a-c), respectively.

**Figure 2:**
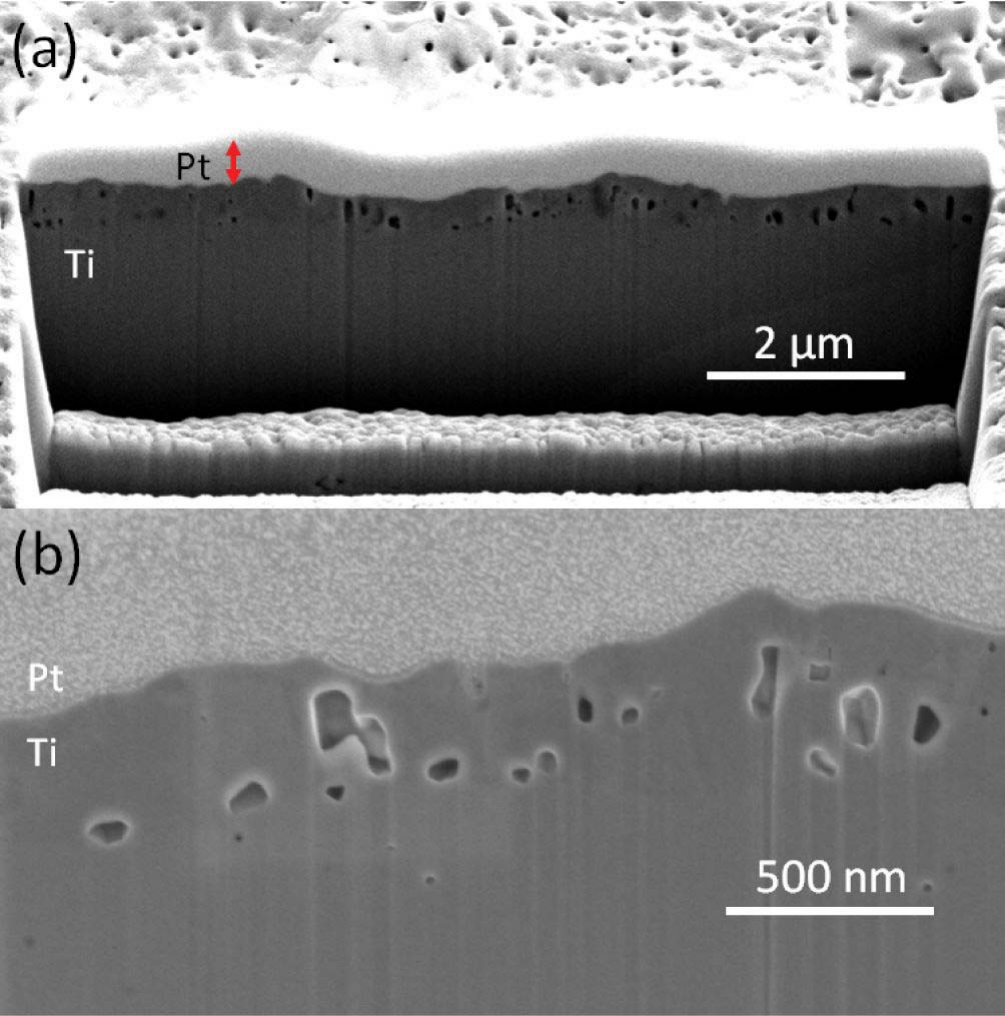
Cross section images of Ti sample (exposed for 10 minutes with surface temperature of 750°C) (iii) taken under low (a) and high magnification (b).

All experimental conditions are listed in table 2.Titanium surfaces were irradiated by low energy (~45 eV) He ions with flux of 2-3x10^23^ m^−2^s^−1^ at surface temperatures of 400, 600 and 750°C for 10 minutes (samples (i), (ii), (iii)). Until around 450°C no clear surface modification was observed. Above that temperature voids start to appear on the surface. As seen in SEM images in (Figure 1.), the areal density of these voids increased with the surface temperature. SEM images were analyzed by the Gwyddion software to specify the mean void size.^37^ There is also a slight increase in the mean diameter of these voids, from 20 nm (88 voids were taken into analysis) to 25 nm (104 voids were taken into analysis) with an increase in surface temperature from 600°C to 750°C. The sample exposed with surface temperature of 750°C was prepared by FIB milling method to get a cross section view. At the beginning of the FIB analysis, a region with an area of 2 µm x 6 µm was coated by Pt to protect that region from the Ga ions. Hence, the etched side gives information about the cross section view of these structures. Voids, which are smaller than 100 nm in diameter, are detected with a wide size distribution underneath the surface (Figure 2.). When these surfaces are compared with other studies in literature, they show a resemblance to those observed after exposures of W and Mo to low energy (~20 eV) He ions.^15^ In that study, an increase of the ion energy to 45 eV led to nanostructure growth. Although the ion energy is around 45 eV in our study, no nanostructure growth is observed.

**Figure 3:**
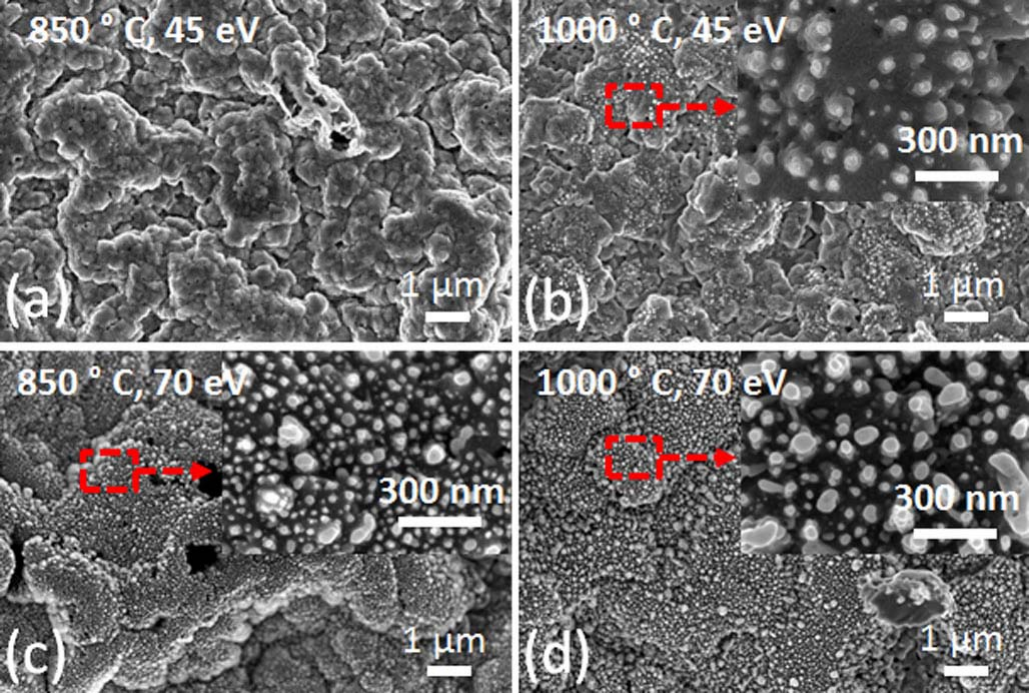
SEM images of samples which are irradiated by He ions with ion energies of 45 eV at surface temperatures of 850°C (0.58 T_m_) and 1000°C (0.66 T_m_) (sample(iv) (a), sample (v) (b)) and ion energies of 70 eV at surface temperatures of 850°C and 1000°C (sample (vi) (c) and sample (vii) (d)).

The surface temperature was increased above 850°C (sample (iv)-(vii), Figure 3a-d). Beyond that temperature, the surface seems to be roughened and reformed. Nanosized structures start to be observed beyond 1000°C at an ion energy of 45 eV, whereas similar nanosized structures are observed at surface temperatures starting from 850°C if the ion energy is increased up to 70 eV. The number of the nanosized structures on a given area tends to decrease by a factor of around 2.5 and the mean diameter is shifted from 36 nm to 53 nm with increase in surface temperature from 850°C to 1000°C (Figure 3c,d.).

### Aluminum

**Figure 4:**
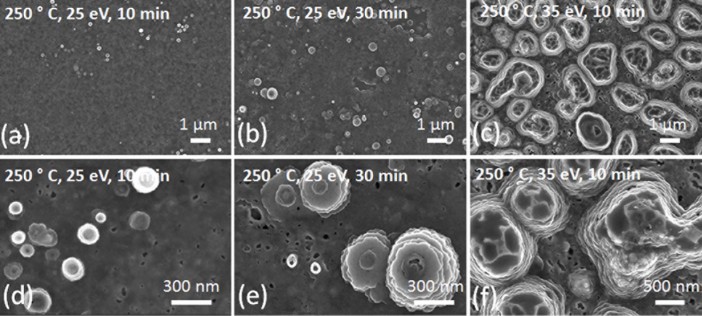
SEM images of Al samples, which are irradiated by He ions with an energy of 25 eV at a surface temperature of 250°C (0.56 T_m_) for 10 minutes and 30 minutes (samples (viii) and (ix)) and with ion energy of 35 eV for 10 minutes (sample (x)), taken under low (a), (b), (c) and high (d), (e), (f) magnification, respectively.

Aluminum surfaces were exposed to He plasma with an ion flux in the range of 3-4x10^23^m^−2^s^−1^ and an ion energy of 25 eV at a surface temperature of 250°C. On these surfaces, randomly located structures besides some voids are detected. (Figure 4a,b) show that for longer exposures the spatial distribution of the structures becomes more homogenous and the average size of voids tends to increase with time.

Since the melting temperature of Al is relatively low (around 660°C) compared to the other metals investigated here, the surface temperature was kept constant at 250°C. Besides the exposure time, the effect of ion energy on surface modification was studied. A significant change in both size and shape of structures is observed with a slight increase in average ion energy, of only 10 eV (see Figure 4c,d). The mean diameter of the structures formed become around 1.4 µm and the voids around 150 nm.

**Figure 5:**
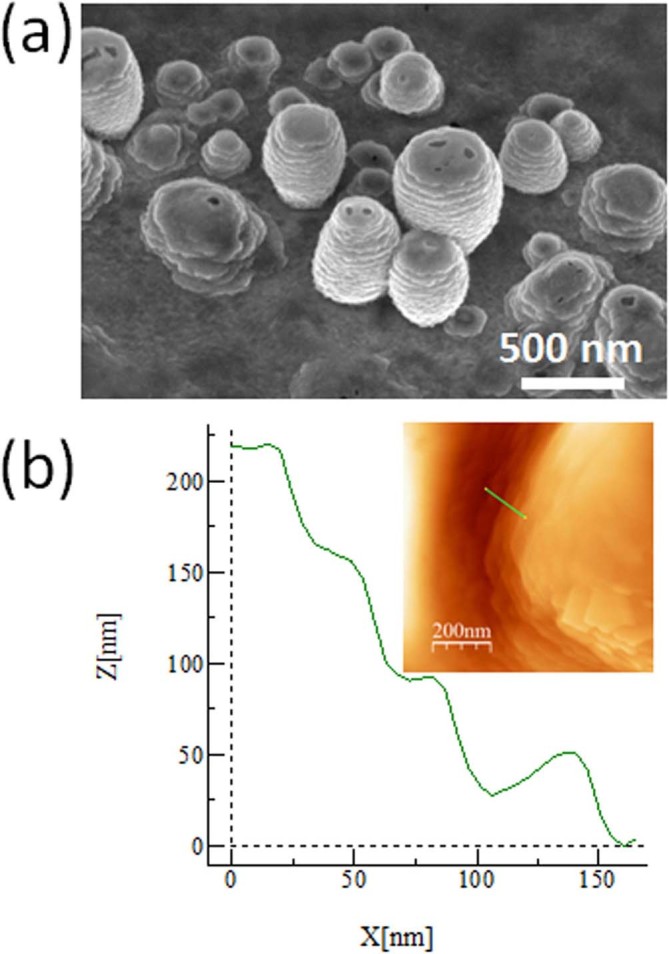
(a) SEM image (30° tilt) taken from sample (viii) (exposure conditions: 25 eV, 250°C, 10 minutes) and (b) AFM image of a structure existing on the same sample.

The tilted SEM image (Figure 5a) shows that surface is covered by individual structures with different heights. Although it is hard to judge from a top view image, the structures seem to be formed of several layers. To gain more insight, AFM measurement has been done on sample (viii). AFM measurement of one of these structures shows that these structures are formed by layers with thickness of around 50 nm (Figure 5b).

**Figure 6:**
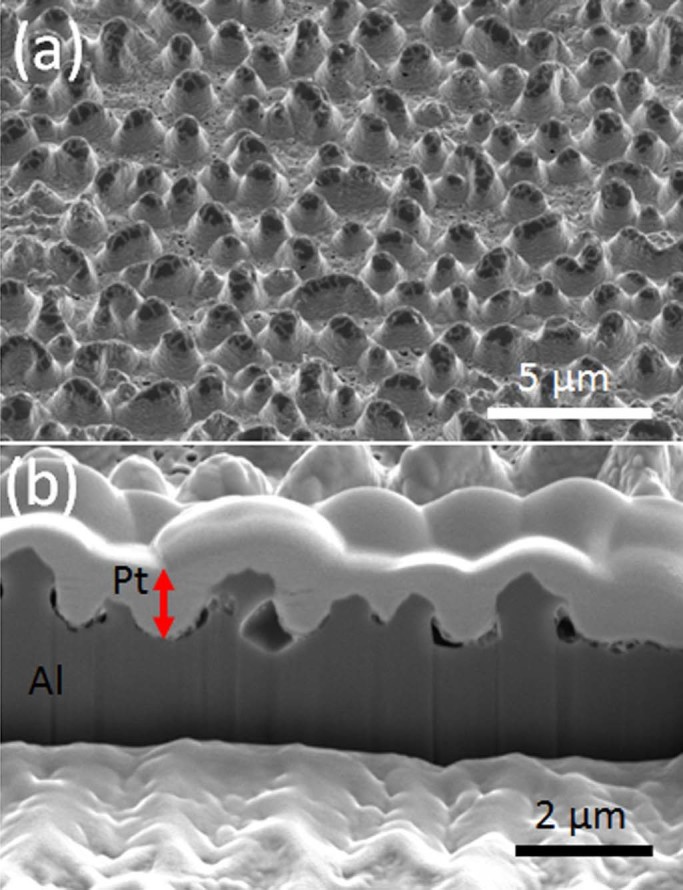
(a) SEM image (52° tilt) of Al surface which was irradiated by He ions with ion energy of 35 eV with surface temperature of 250°C (sample (x)) and (b) a cross sectional image which was taken from the region seen in (a) (white layer seen on top is Pt which was coated during FIB milling).

In order to clarify the surface modification mechanism, cross sectional images were taken from the sample prepared by FIB milling method. (Figure 6) exhibits the effect of He ions on surface modification with the presence of voids underneath the surface of the sample (x).

### Copper

**Figure 7:**
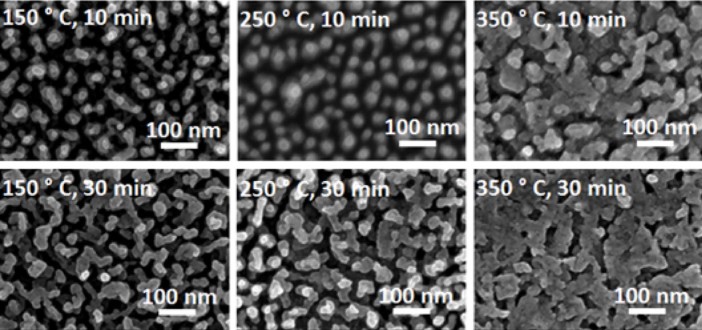
Evolution of Cu nanostructures with surface temperature (150-350°C, 0.31-0.46 T_m_) and exposure time (10, 30 minutes).

Copper surfaces were irradiated by He ions with flux of 5-7x10^23^m^−2^s^−1^ and at an ion energy of 25 eV. The surface temperature during these exposures was kept in the intermediate temperature range (0.3 *T_m_*< *T* < 0.5 *T_m_*) and exposures were repeated for two different durations, 10 and 30 minutes. As seen in (Figure 7), no significant change in the form of nanostructures can be observed for different exposure times. With the increase in surface temperature, individual structures tend to enlarge and then connect to each other for both exposure times.

**Figure 8:**
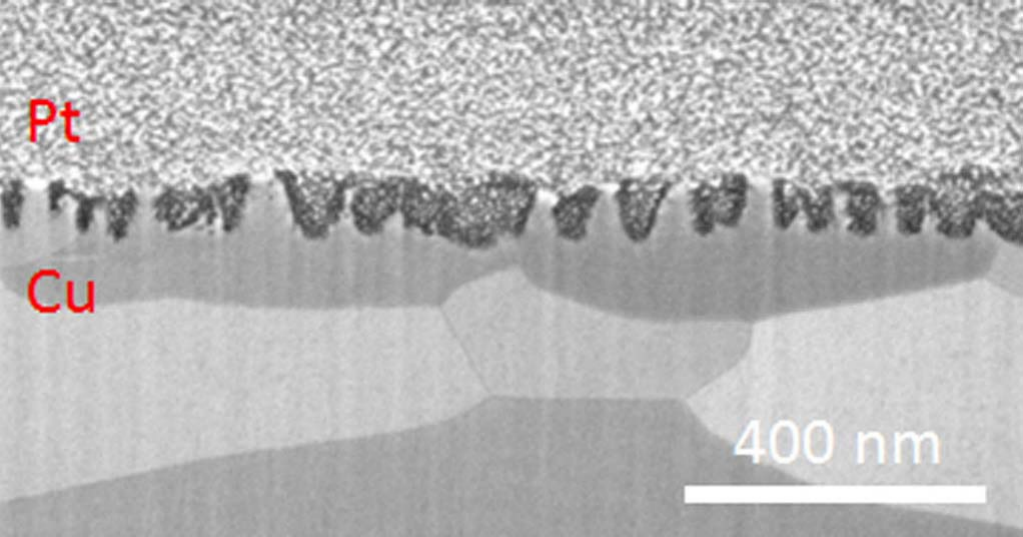
Cross sectional image of Cu surface, which was exposed to helium plasma with surface temperature of 250°C for 10 minutes (sample (xii)) prepared by FIB milling method.

Similarly to the Al surfaces, sample (xii) was prepared by FIB milling and a cross sectional image was taken. As seen in (Figure 8), a nanostructured layer with a thickness of around 100 nm is observed without any trace of He-induced voids.

**Figure 9:**
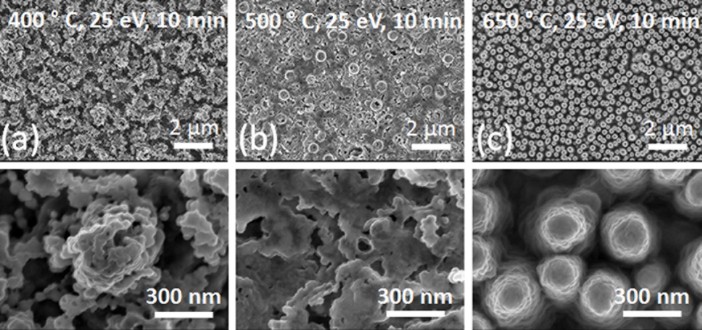
SEM images of surfaces which are irradiated by He ions with ion energy of 25 eV at surface temperatures of (a) 400°C (0.50 T_m_) (sample (xiv)), (b) 500°C (0.57 T_m_) (sample (xv)) and (c) 650°C (0.68 T_m_) sample (xvi). Images in the lower row are taken under higher magnification.

**Figure 10:**
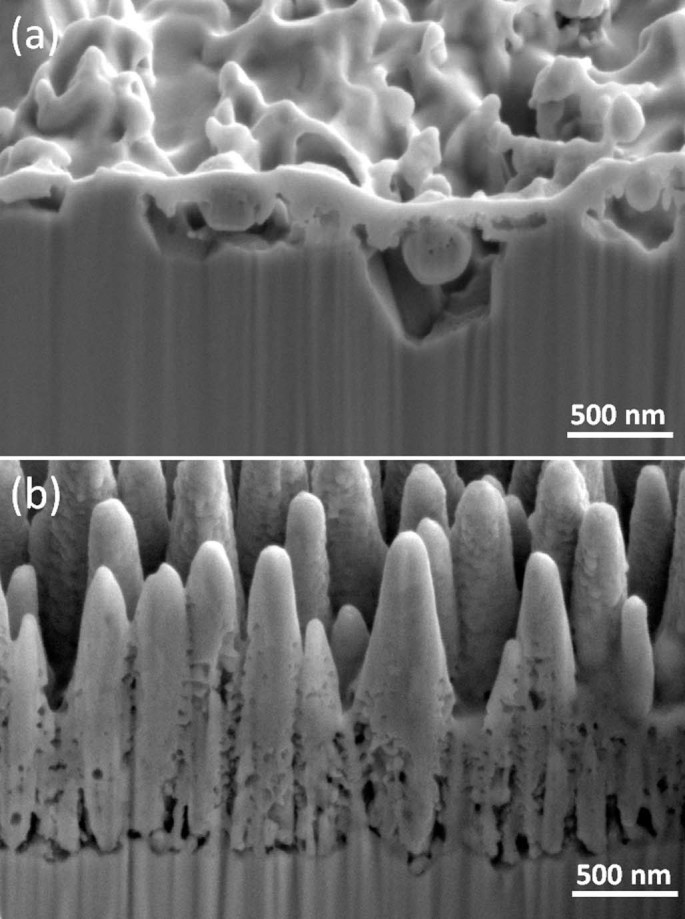
Cross section images of samples, which are exposed to helium plasma with surface temperatures of (a) 500°C (sample (xv)) and (b) 650°C (sample (xvi)).

Copper surfaces were exposed at higher temperatures, above 0.5 *T_m_* resulting in significant changes in morphology. The structures observed at the highest temperature for our range resemble the ones on Al, but in this case they are homogenously distributed and almost in the same size, around 240 nm in diameter (Figure 9). (Figure 10) shows the cross sectional views of samples (xv) and (xvi). In contrast with the samples exposed with lower surface temperature (<400°C), voids are clearly detected underneath the surface. The homogenously distributed structures, which are formed after exposure at 650°C, are clearly visible on the cross sectional images in (Figure 10b) and seem to have pillar-like shape.

## Discussion

In order to observe the variation in efficacy of He ion induced surface modification of metals with different crystal structures, we investigated Ti, which has hcp type crystal structure, and Al and Cu, which have fcc type crystal structure, under similar irradiation conditions. Our results show that He ions could penetrate and form voids underneath the surface in all metals that we worked with. However, in terms of the resulting surface modifications different metals behave differently. Ti exposures do not show any nanostructure growth on the surface, which seems consistent with the expectations considering its densely packed crystal structure or low population of interstitials which mostly mediate effective He diffusion in the metal. Kajita et al. recently published an extensive study on the surface modification of titanium after He plasma exposure.^38^ Significant changes in surface morphology have been observed with slight differences in irradiation conditions, such as ion energy. Most of their exposures are conducted at higher ion energy (>70 eV) than in our experiments. For that ion energy range, cone like structures are observed on the surface due to the enhanced effect of physical sputtering. For slightly lower ion energies (~50 eV),^38^ void formation has been reported on the surface, which is quite similar to what is observed in our experiments at similar surface temperatures and ion energy value (~45 eV). Besides that, the morphology obtained at 927°C (Figure 2 in Ref. 38.) is similar to the surfaces obtained at 850°C in our experiments. In that temperature range, the surface seems to be roughened and nanosized structures are formed.

Once the survey shifted to metals with lower mass compared to tungsten and molybdenum, other effects have to be invoked in the explanation of surface modifications under low energy He ion irradiations. Our previous work and also other studies suggested physical sputtering as an additional mechanism which contributes to the surface modification.^16^,^17^,^39^ In studies conducted on tungsten and molybdenum the sputtering yield is considered as negligible, since the ion energy in those studies is typically well below the threshold energy for physical sputtering. In our study, the mass loss is measured by weighing the samples before and after the plasma exposure. The sputtering yield is then determined by using the following expression: 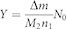
where Δ*m* is mass loss, *M_2_* - atomic mass of metal of interest, *n_1_* - number of He ions reaching the surface and *N_o_* - Avogadro's number.

**Figure 11:**
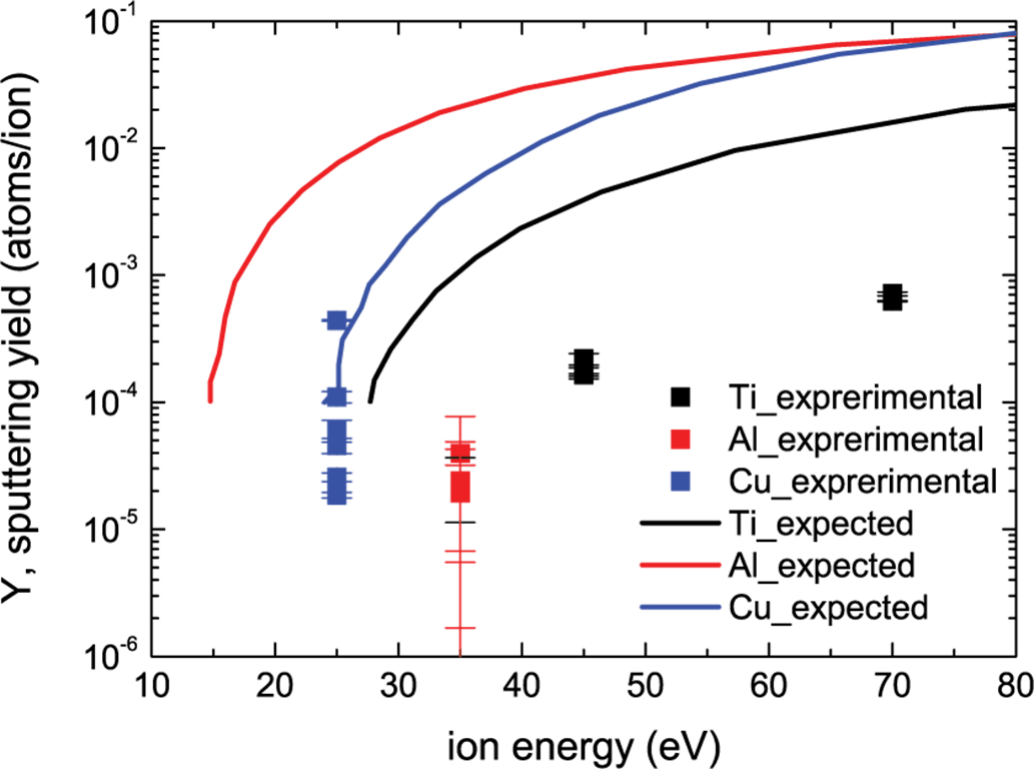
Sputtering yield of Ti, Al and Cu regarding to our mass loss measurements and literature values^41^.

The sputtering yields measured here (taken on samples (i)-(xix)) are compared with the fit to several calculated values.^40^ (Figure 11) shows that our values are around one order of magnitude below the expected sputtering yields of the metals that we worked with. It is worth to note that the calculations are usually done by assuming a nearly flat surface, i.e. the effect of surface morphology on the sputtering yield is not taken into account. The curvature dependent sputtering has already been proposed in Sigmund’s theory.^41^ Based on that theory, an analytical formula for the morphology dependent sputtering yield is developed and accordingly a decrease in sputtering yield is predicted with the development of surface morphology.^42^ The morphological change induced here by He ion irradiation is more complex than a symmetrical structure, hence one would expect to have enhanced deviation between calculated and experimental data. The variation in the sputtering yield of flat and modified surface has been reported after a similar experimental study as well. Nishijima *et al.* have shown that the sputtering yield of nanostructured tungsten derived from mass loss measurements are around one order of magnitude lower than the values calculated by TRIM code.^43^ The deviation in the calculated and experimental results is in agreement with our results. Different from the other metals, the sputtering yield for Al that we obtained from our experiments is around two orders of magnitude lower than the literature values. Since Al is quite reactive and native aluminum oxide (most likely, Al_2_O_3_) has higher binding energy than Al, we would expect a decrease in sputtering yield for samples (viii)-(x).

**Figure 12:**
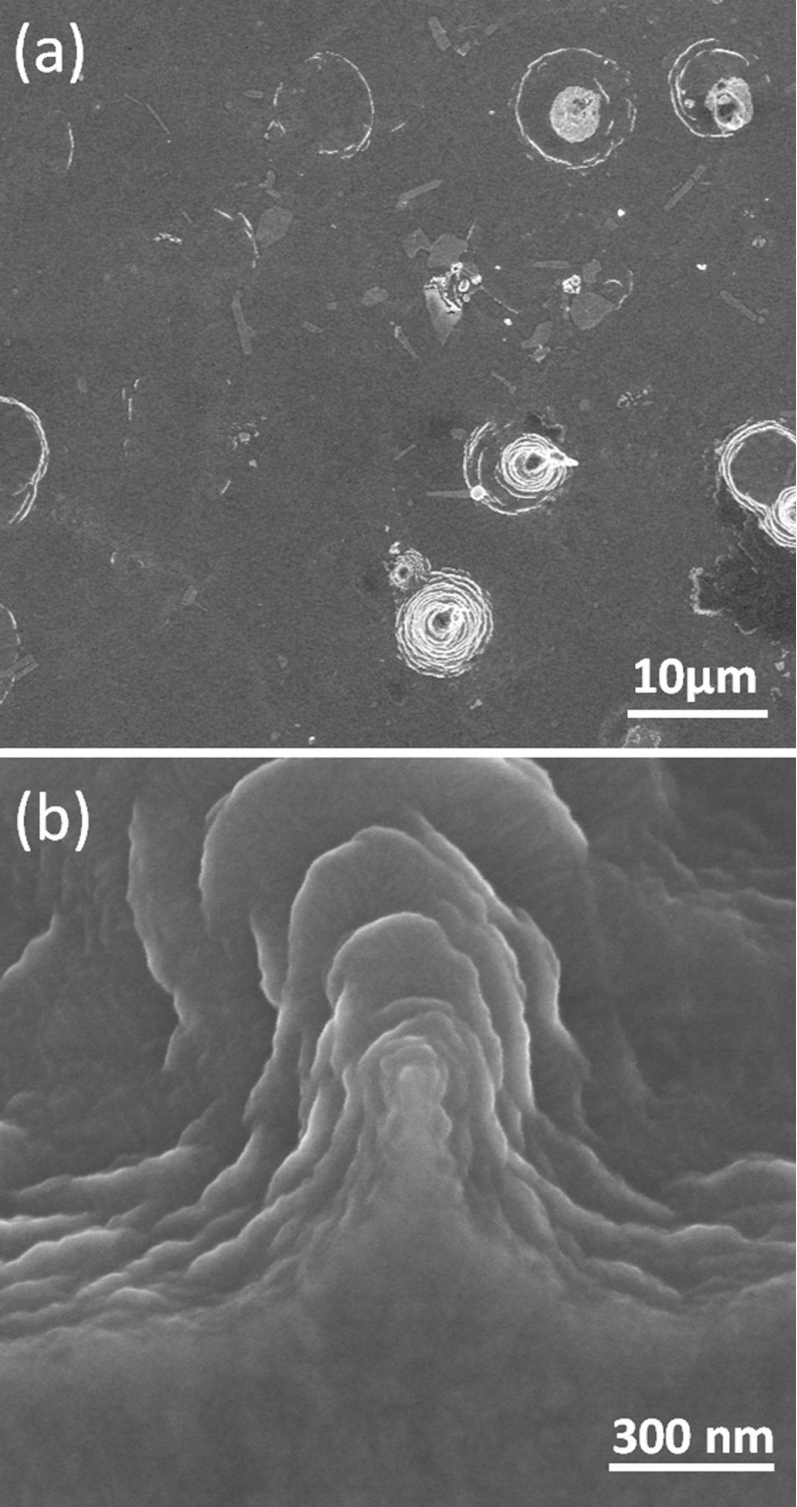
SEM images of Al surface after Ar plasma exposure taken under low (a) and high (b) magnification.

The structures on Al and Cu surfaces show similarity with the self-organized nanopatterns and nanodots obtained by ion beam sputtering.^44^,^45^ The formation of these patterns is explained as a result of interplay between ion sputtering, which induces surface roughening, and surface diffusion, which induces smoothing.^44^ To investigate the role of physical sputtering on the observed morphology changes, several studies have been conducted on W and Ti surfaces exposed to both Ar and Ne plasma for W, and Ne plasma for Ti. In neither of those cases, nanostructure formation or bubble growth beneath the surface were detected.^38^,^46^ Similarly, Al surfaces were exposed to Ar ions and (Figure 12) shows that Ar ions induce no surface modification except for some sparse structures whose layered structure resembles the nanostructures formed under He plasma. However, there is no significant similarity between surfaces after He and Ar plasma exposures, namely neither homogenously located nanostructures nor voids are detected. Hence, the surface modifications that we observe on Al and Cu cannot be only attributed to physical sputtering caused by any ion species. The effect of He clustering and consequently void formation on surface modification is clearly seen on Al and Cu surfaces. Both He ion irradiation and physical sputtering would be considered as effective factors in the morphology changes of Cu and Al.

Titanium, aluminum and copper surfaces were exposed to pure He plasmas to study the associated morphology changes. Different surface modifications were observed among these metals. The experimental studies show that it is rather hard to rely on a single material property in order to predict the behavior of metals after helium plasma exposures. The effect of physical sputtering on surface modification is clearly seen for Al and Cu surfaces. Homogenously distributed nano pillars are observed on these metals. Any significant surface modification could not be observed for Ti, which might be resulted because of its closely packed structure and low sputtering yield. Once the nano pillars formed on Cu surfaces are oxidized, they could be of interest for further energy applications, such as electrochemical reductions of CO_2_ and photoelectrochemical water splitting, because of their homogenous distribution and high aspect ratios.^47^

## Author Contributions

The experiments were planned by I.T. and G.d.T. and conducted by I.T. SEM measurements and FIB milling were performed by D.M. The results were discussed by I.T., M.C.M.v.d.S. and G.d.T. Manuscript was written by I.T. with improvements from G.d.T., M.C.M.v.d.S. and L.M.

## Figures and Tables

**Table 1:** Formation, migration energies of He interstitial and substitutional detrapping energies for W, Mo, Fe, Ti, Al and Cu.

	Formation energy (in eV) of a He interstitial	Migration energy (in eV) of a He interstitial	Substitutional detrapping energy (in eV)	Reference [26-32]
W (bcc)	5.47	0.24	4.75	[26,27]
	5.91	0.29	4.42	
Mo (bcc)	4.91	0.23	4.19	[26,27]
	4.97	0.3	3.87	
Fe (bcc)	5.36	0.17	3.98	[26]
			3.75	
Ti (hcp)	2.67	0.34		[28]
Al (fcc)	1.25	0.13		[29-32]
	1.32	0.16		
	3.02			
Cu (fcc)	2.03	0.45	1.88	[26]

**Table 2:** Experimental conditions.

	Metal	Ion energy (eV)	Surface temperature (°C)	T/T_m_	Time (min)
i	Ti (hcp)	45	400	0.35	10
ii		45	600	0.45	10
iii		45	750	0.53	10
iv		45	850	0.58	10
v		45	1000	0.66	10
vi		70	850	0.58	10
vii		70	1000	0.66	10
viii	Al (fcc)	25	250	0.56	10
ix		25	250	0.56	30
x		35	250	0.56	10
xi	Cu (fcc)	25	150	0.31	10
xii		25	250	0.39	10
xiii		25	350	0.46	10
xiv		25	400	0.50	10
xv		25	500	0.57	10
xvi		25	650	0.68	10
xvii		25	150	0.31	30
xviii		25	250	0.39	30
xix		25	350	0.46	30
